# The Organophosphate Paraoxon and Its Antidote Obidoxime Inhibit Thrombin Activity and Affect Coagulation *In Vitro*

**DOI:** 10.1371/journal.pone.0163787

**Published:** 2016-09-30

**Authors:** Valery Golderman, Efrat Shavit-Stein, Ilia Tamarin, Yossi Rosman, Shai Shrot, Nurit Rosenberg, Nicola Maggio, Joab Chapman, Arik Eisenkraft

**Affiliations:** 1 Laboratory for Neurological Research, Sheba Medical Center, Tel HaShomer, Israel; 2 Department of Neurology, the Chaim Sheba Medical Center, Tel HaShomer, Israel; 3 Department of Hematology, Coagulation Laboratory, Sheba Medical Center, Tel HaShomer, Israel; 4 Surgeon General Headquarters, Israel Defense Force Medical Corps, Tel Hashomer, Ramat Gan, Israel; 5 Sackler Faculty of Medicine, Tel Aviv University, Tel Aviv, Israel; 6 Department of radiology, MedStar Georgetown University Hospital, Washington DC, United States of America; 7 Department of Neurology, Sackler Faculty of Medicine and Sagol School of Neuroscience, Tel Aviv University, Tel Aviv, Israel; 8 Robert and Martha Harden Chair in Mental and Neurological Diseases Sackler Faculty of Medicine, Tel Aviv University, Tel Aviv, Israel; 9 NBC Protection Division, IMoD, Hakyria, Tel Aviv, Israel; 10 Institute for Research in Military Medicine, the Hebrew University of Jerusalem, Jerusalem, Israel; Weizmann Institute of Science, ISRAEL

## Abstract

Organophosphates (OPs) are potentially able to affect serine proteases by reacting with their active site. The potential effects of OPs on coagulation factors such as thrombin and on coagulation tests have been only partially characterized and potential interactions with OPs antidotes such as oximes and muscarinic blockers have not been addressed. In the current study, we investigated the *in vitro* interactions between coagulation, thrombin, the OP paraoxon, and its antidotes obidoxime and atropine. The effects of these substances on thrombin activity were measured in a fluorescent substrate and on coagulation by standard tests. Both paraoxon and obidoxime but not atropine significantly inhibited thrombin activity, and prolonged prothrombin time, thrombin time, and partial thromboplastin time. When paraoxon and obidoxime were combined, a significant synergistic effect was found on both thrombin activity and coagulation tests. In conclusion, paraoxon and obidoxime affect thrombin activity and consequently alter the function of the coagulation system. Similar interactions may be clinically relevant for coagulation pathways in the blood and possibly in the brain.

## Introduction

Organophosphates (OPs) are a major cause of threat on the health of civilians and military personnel due to their use as pesticides as well as chemical weapons in war or in terror attacks [1]. By inhibiting acetylcholinesterase (AChE), OPs lead to a cholinergic crisis both in the central and peripheral nervous system [2].

The immediate clinical signs and symptoms of OP poisoning include hypersecretions and fasciculations, miosis, tremor, convulsions, paralysis, coma and finally death primarily due to respiratory failure [3], while late symptoms result from brain damage and include neurological and psychiatric sequelae [4]. The standard treatment of OP poisoning consists of timely administration of atropine and oximes. Atropine, by blocking the action of acetylcholine at muscarinic receptors, reduces the amount of secretions. Oximes such as obidoxime break the bond between the OP and the enzyme and reactivate it [4].

Several blood clotting factors have been found to be sensitive to OPs [5–7], however, the exact relationship of such effect was not thoroughly investigated.

In the current study, we sought to examine the possible interactions between thrombin, a blood coagulation factor, with the OP paraoxon and obidoxime. We found that both paraoxon and obidoxime could interfere with thrombin activity, ultimately impairing the coagulation cascade. Our data also show that coagulation is prolonged in the presence of paraoxon and its antidote obidoxime. Future investigations are needed in order to better identify the clinical implications of such phenomena.

## Materials and Methods

### Materials

Synthetic peptide substrate Boc-Asp(OBzl)-Pro-Arg-AMC (I-1560) was purchased from Bachem. Bestatin was purchased from Cayman chemical company (70520). Prolyl endopeptidase inhibitor was purchased from Calbiochem (537011). Obidoxime chloride 250 mg/ml was purchased from MerckSerono. Bovine thrombin, paraoxon and atropine were purchased from Sigma (T4648, N-12816 and A0257 respectively). Paraoxon dilution was performed in a chemical hood. Paraoxon was first diluted with propylene glycol to 50 mg/ml, and next diluted with saline to 1.36 mg/ml. All paraoxon contaminated instruments and dishes were neutralized using 1.7 g/L sodium dichloroisocyanurate [8]. Neutralized waste was stored in waste containers and disposed according to regulations by the sanitation department of the Sheba Medical Center.

### Thrombin like activity

Thrombin activity was measured as previously described [9,10]. Briefly, using a fluorometric assay, we quantified the cleavage of the synthetic peptide substrate Boc-Asp(OBzl)-Pro-Arg-AMC (excitation at 355–380 nm, emission measured at 440–460 nm). The reaction was carried out in a 96-well microplate. The substrate (5 μl final concentration 14 μM) was added to the reaction buffer (50 mM TRIS/HCL, pH = 8, 0.15 M NaCL, 1 mM CaCl2) and 0.1% BSA (total volume per well 100 μl). In order to eliminate the effect of aminopeptidases and endopeptidases all the reactions were performed in the presence of 0.1 mg/ml bestatin and 2 μM prolyl endopeptidase inhibitor. For calibration, known concentrations of bovine thrombin were used in the same assay. The assay was performed in four different settings: 1. Constant concentration of thrombin (0.05 U/ml) and varying concentrations of paraoxon from 0.5 mM to 500 nM. 2. Constant concentration of thrombin (0.05 U/ml) and varying concentrations of obidoxime from 3 mM to 3nM. 3. Constant concentration of thrombin (0.05 U/ml) and varying concentrations of atropine from 0.9 mM to 3 μM. 4. Constant concentration of thrombin (0.05 U/ml) and paraoxon (5μM), and varying concentrations of obidoxime (3 mM–3 nM).

### Coagulation in plasma

Human citrated plasma from healthy donors was purchased from Instrumentation Laboratory (normal control Assayed, 0020003110) as lyophilized human plasma containing buffer, stabilizers and preservatives. It was reconstituted using standard laboratory methods. The product is designed as a normal control for monitoring coagulation assays: PT, APTT, TT, fibrinogen, single factors, protein C and S etc. The plasma was exposed *in vitro* to 0.5 mM paraoxon, 30 mM obidoxime as well as paraoxon and obidoxime combined together, for ten minutes. Next, prothrombin time [9], activated partial thromboplastin time (APTT) and thrombin time (TT) were measured. All tests were carried out with ACLTOP^®^ 500 analyzer (Instrumentation Laboratory).

## Results

### The effect of paraoxon on thrombin activity *in vitro*

**Fig 1:**
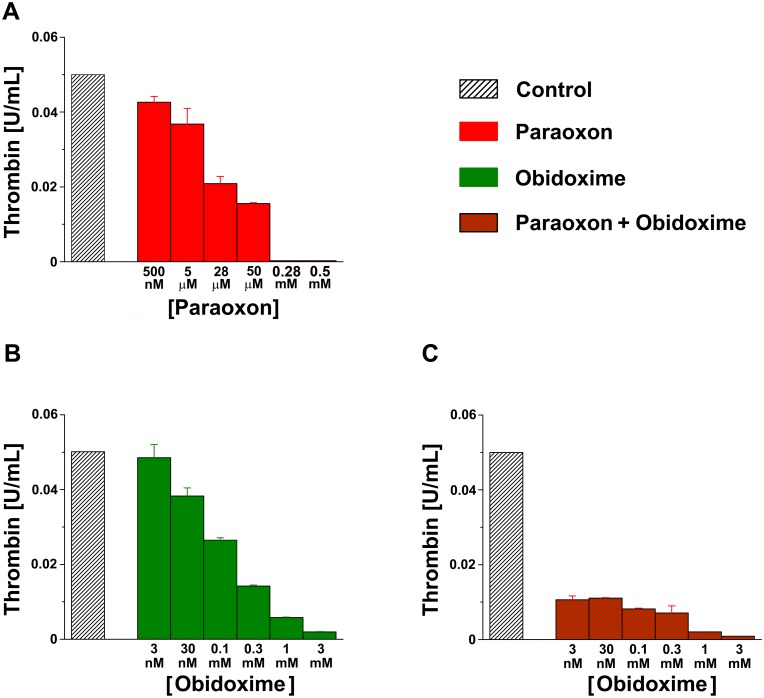
Paraoxon and obidoxime inhibit thrombin activity *in vitro*. **(A)** Varying concentrations of paraoxon (500 nM, 5 μM, 28 μM, 50 μM, 0.28 mM and 0.5 mM) inhibit thrombin activity. **(B)** Obidoxime inhibits thrombin activity at varying concentrations. In the presence of 3 nM obidoxime there is no significant change in thrombin activity. **(C)** Thrombin activity is significantly inhibited when obidoxime is applied together with paraoxon.

We tested whether paraoxon and its accepted antidotes obidoxime and atropine may affect thrombin activity *in vitro*. For this purpose, we adapted a methodology originally developed in our lab [11], and exposed each of these compounds at variable concentrations to constant levels of thrombin (0.05 U/ml). The plate was kept on ice for 10 minutes, before substrate addition. Substrate cleavage monitored for an hour at room temperature. Fig 1A depicts the effects of varying concentrations of paraoxon (0.5 mM–500 nM) on thrombin activity. Paraoxon inhibited thrombin activity in a dose-dependent manner with the highest effect found at concentration of 0.5 mM paraoxon (0.015±2.6^e-4^). A single way ANOVA taking into account the different concentrations as a factor for comparison revealed a significant effect of all tested concentrations (F = 117.2, p<0.001, n = 3 for each concentration tested) with the highest effect shown at high concentrations as detected by a *post hoc* Bonferroni *t* test. Based on this data, the constant of inhibition (Ki) of paraoxon is 9.6 μM (using Cheng & Prusoff, 1973 method for Ki determination [12]).

### Obidoxime inhibits thrombin activity *in vitro*

We evaluated the effects of obidoxime on thrombin activity by adding varying concentrations of obidoxime (3 mM– 3 nM) to a constant concentration of thrombin (0.05 U/ml). As described previously, the plate was kept on ice and monitored for an hour after substrate addition. This range of obidoxime concentrations was chosen based on a paraoxon poisoning model in rodents, where the standard treatment consists on the administration of 30 nM obidoxime. Strikingly, obidoxime also caused a dose dependent inhibition of thrombin activity (0.03±0.002; Fig 1B). A single way ANOVA taking into account the different concentrations as a factor for comparison revealed a significant effect of some tested concentrations (F = 131.7, p<0.001, n = 3 for each concentration tested) with the highest effects detected at 3 mM obidoxime and the lowest at concentrations of 30 nM (p<0.001 and p<0.05 with *post hoc* Bonferroni *t* test, respectively). Based on this data, the Ki of obidoxime is 0.05 μM (using Cheng & Prusoff, 1973 method for Ki determination [12]). For the purposes of this preliminary report, we have measured the concentration dependence of paraoxon on thrombin activity and estimated an apparent IC-50 and binding constant, Ki. Organophosphate inhibitors, including paraoxon, are known to inhibit thrombin progressively by formation of a covalent bond with the catalytic serine [6,7]. We recognize that a rigorous analysis of inhibitor-enzyme interaction will require a more complete kinetic analysis, for example measurement of the time-dependent bimolecular rate constant of inhibition. Nevertheless, several previous studies have found it useful to report an apparent IC-50 or Ki as we do herein to rank-order or compare inhibition of thrombin by various organophosphates [5,13].

### The effect of atropine on thrombin activity *in vitro*

We continued the experiment with an additional antidote—atropine. Varying concentrations of atropine (0.9 mM, 0.3 mM, 90 μM, 30 μM, 9 μM, 3 μM) were added to a constant concentration of thrombin (0.05 U/ml), however no effect of atropine on thrombin activity *in vitro* was detected (data is available in S1–S5 Tables).

### The combination of paraoxon and obidoxime synergistically inhibits thrombin activity *in vitro*

In order to examine the combined effect of paraoxon and obidoxime on thrombin activity *in vitro*, we exposed constant concentrations of both thrombin (0.05 U/ml) and paraoxon (5 μM) to varying concentrations of obidoxime (3 mM–3 nM). The plate was kept on ice for 10 minutes. After substrate addition, the cleavage was monitored for an hour at room temperature. When combined together the two substances synergistically inhibited thrombin activity (Fig 1C). A single way ANOVA taking into account the different concentrations as a factor for comparison (F = 339.06, p<0.001, n = 3 for each concentration tested) followed by a *post hoc* Bonferroni t Test revealed a significant effect mainly at 3 mM concentrations (p<0.0001).

### Paraoxon and obidoxime affect coagulation tests in plasma

**Fig 2:**
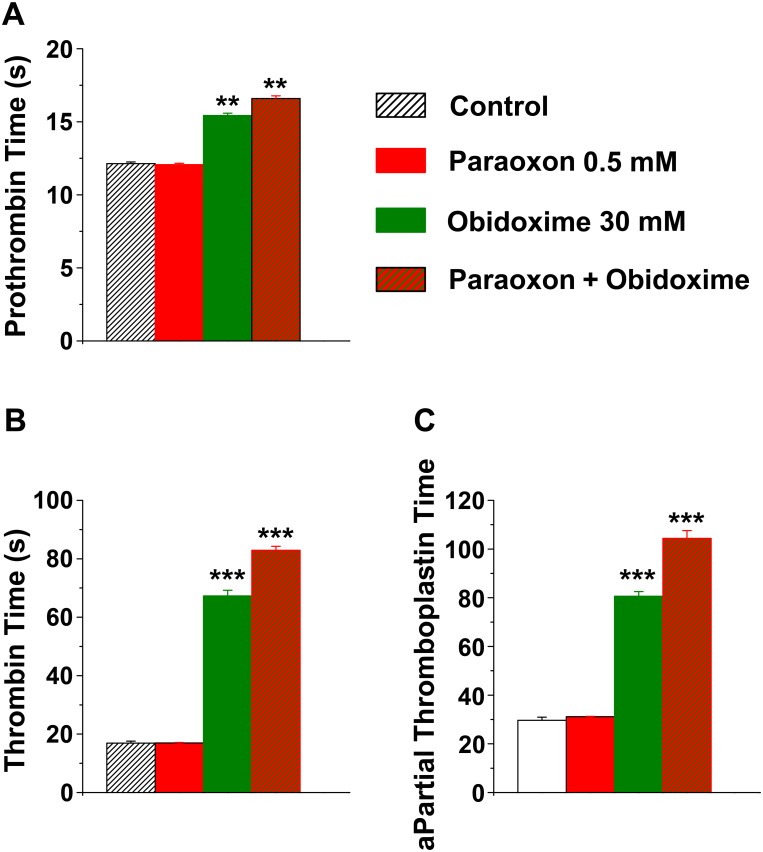
The effects of paraoxon and obidoxime on coagulation. **(A)** Prothrombin time, **(B)** Thrombin Time (TT) and **(C)** activated partial thromboplastin time (APTT) are significantly prolonged by obidoxime as well as by its combination with paraoxon.

In order to understand whether paraoxon and obidoxime could influence the coagulation system because of their ability to inhibit thrombin activity, we examined their effects on prothrombin time [14], activated partial thromboplastin time (aPTT) and thrombin time (TT) in plasma. PT assesses the extrinsic and common pathways of coagulation with prolonged PT being caused by anticoagulants. aPTT assesses the intrinsic and common pathways of coagulation with prolonged aPTT being caused by anticoagulants and also a direct thrombin or factor Xa inhibition. TT measures the final step of coagulation, the conversion of fibrinogen to fibrin. Prolonged TT is caused by thrombin inhibitors [5]. These experiments were conducted as previously shown: we first tested the effects of paraoxon and obidoxime alone and next tested both substances together. At high concentrations, obidoxime alone significantly affected all three coagulation tests. Specifically, a significantly prolonged PT (15.4±0.16 vs. 12±0.12 of control; n = 4, F = 249.2, p<0.01; Fig 2a), TT (67.3±1.9 vs. 16.9±0.7 of control; n = 4, F = 700.46, p<0.001; Fig 2b) and aPTT (80.6±1.8 vs. 29.6±1.3 of control; n = 4, F = 327.44, p<0.01; Fig 2c), was found in the presence of 30 mM obidoxime. The combination of 0.5 mM paraoxon and 30 mM obidoxime was even more effective in increasing all coagulation measurements. Indeed, under these conditions PT, TT and aPTT were 16.6±0.17, 83±1.36 and 104.3±3.18, respectively (n = 4 for each condition). Under the latter circumstances, while 0.5 mM paraoxon alone was not able to significantly affect the coagulation tests, in combination with 30 mM obidoxime it significantly affected all three tests.

## Discussion

In the current study, we investigated the *in vitro* interactions between thrombin, paraoxon and obidoxime as well as atropine. Thrombin, a serine protease, play fundamental roles in the coagulation system as well as in the immune and nervous systems [15–17]. While it has long been known that paraoxon inhibits esterases such as AChE and BuChE [15–17], little is known about the interaction between thrombin and paraoxon alone or in combination with its antidotes, atropine and obidoxime.

Here, we report that paraoxon inhibits thrombin activity *in vitro* in a concentration-dependent manner. Surprisingly, its antidote obidoxime shared these effects, while atropine did not. This latter finding points to a specific effect of both paraoxon and obidoxime on the thrombin activity assay. Notwithstanding, the combination of paraoxon and obidoxime resulted in even more striking effects. When applied together, they synergistically halted thrombin activity such that lower concentrations of obidoxime were about three times more potent when applied together with paraoxon than alone. Based on that, however taking into account the very high tested concentrations, it seems as if paraoxon and obidoxime may influence coagulation as they increased PT, aPTT and TT in human plasma.

In the past, clinical investigations reported prolonged PT and consequent coagulation abnormalities following OP exposure already in the acute phase of poisoning [18]. Whether a coagulation disorder induced by OPs may constitute part of the chronic sequelae of the poisoning is currently unknown. Despite the major implications that coagulation disturbances may have on patients, currently there are no clear guidelines that address this issue in the poisoned patient. Previous clinical evidence [19] together with our findings call for further investigations into such interactions as to be able to implement clear plans for the screening of coagulation disorders in poisoned individuals. This policy may have an influence on the prevention of possible acute as well as chronic consequences.

The mechanism by which OPs impair coagulation is unknown. Previous suggestions have pointed to a possible interaction between OPs and the processes leading to vitamin K synthesis [18]. This could result in the dysfunction of vitamin K-dependent coagulation factors [5]. Indeed, the injection of vitamin K in a OP-poisoned patient resulted in the successful arrest of acute haemorrhage [20]. Our data, however, points to an even more complex interaction between OPs and the coagulation system. Indeed, we show that paraoxon is able to directly block thrombin activity *per se* besides its reported role as a vitamin K synthesis inhibitor. A possible mechanism may involve the ability of paraoxon to directly interact and thus inhibit proteases [20]. Further experiments investigating the chemical reactions between paraoxon and thrombin's active site may shed more light on this phenomenon.

Besides the effects of paraoxon, the finding that obidoxime, an OP antidote, is also able to inhibit thrombin activity *in vitro* with an enhanced, synergistic effect in the presence of paraoxon, is surprising. Such effect could be the result of the formation of Mono(diethylphosphoryl) obidoxime (DEP-obidoxime). The low temperature provided the stable condition for DEP-obidoxime formation as described by Kiderlen et al. [20]. A possible explanation for a greater inhibition in plasma coagulation tests may be the activity of Paraoxonase 1 (PON1) [20]. Indeed, the combination of paraoxon and obidoxime may result in an early saturation of PON1 activity thus influencing the coagulation parameters. Further experiments are needed in order to prove this speculation.

We should bear in mind that obidoxime has other effects besides its reactivation properties. Our data may point to the possibility that the combination of paraoxon and obidoxime may worsen the OP-induced coagulation disorder, and an adjusted clinical approach may be needed in order to effectively manage it.

In conclusion, our data shows that paraoxon and obidoxime affect thrombin activity and consequently alter the function of the coagulation system. Further investigations are needed in order to understand the clinical consequences of such effects in order to adapt currently accepted practices for the effective and comprehensive management of all aspects of OP poisoning.

## Supporting Information

S1 Table Average thrombin activity in the presence of varying concentrations of paraoxon. Average thrombin activity and standard deviation as calculated from three different measurements of thrombin activity assay.
(PDF)

S2 Table Average thrombin activity in the presence of varying concentrations of toxogonin. Average thrombin activity and standard deviation as calculated from three different measurements of thrombin activity assay.
(PDF)

S3 Table Average thrombin activity in the presence of 5uM paraoxon and varying concentrations of toxogonin. Average thrombin activity and standard deviation as calculated from thee different measurements of thrombin activity assay.
(PDF)

S4 Table Average thrombin activity in the presence of varying concentrations of atropine. Average thrombin activity and standard deviation as calculated from six different measurements of thrombin activity assay.
(PDF)

S5 Table Average TT, PT and APTT in the presence of paraoxon and toxogonin. Average times and standard deviation as calculated from three different measurements.
(PDF)
