# Supplementary material for: The Organophosphate Paraoxon and Its Antidote Obidoxime Inhibit Thrombin Activity and Affect Coagulation In Vitro
Source: PLoS One. 2016 Sep 30;11(9):e0163787. doi: 10.1371/journal.pone.0163787 (PMC5045196; doi:10.1371/journal.pone.0163787)
Supplement: S1 Table — Average thrombin activity and standard deviation as calculated from three different measurements of thrombin activity assay. (PDF) [file pone.0163787.s001.pdf]

S1 Table

Paraoxon	Average thrombin activity (U/ml)	Standard deviation
0.5 mM	0.000262131	7.04878E-05
0.28 mM	0.000279692	2.61537E-05
50 µM	0.0155275	0.000453569
28 µM	0.02087	0.00334807
5 µM	0.0368	0.007278638
500 nM	0.042635	0.002662926
Control	0.05	0
