# Supplementary material for: The Organophosphate Paraoxon and Its Antidote Obidoxime Inhibit Thrombin Activity and Affect Coagulation In Vitro
Source: PLoS One. 2016 Sep 30;11(9):e0163787. doi: 10.1371/journal.pone.0163787 (PMC5045196; doi:10.1371/journal.pone.0163787)
Supplement: S2 Table — Average thrombin activity and standard deviation as calculated from three different measurements of thrombin activity assay. (PDF) [file pone.0163787.s002.pdf]

S2 Table

Toxogonin	Average thrombin activity (U/ml)	Standard deviation
3 mM	0.00196	2.91285E-05
1 mM	0.005799345	5.45641E-05
0.3 mM	0.014231	0.00046213
0.1 mM	0.0265025	0.001056986
30 nM	0.038295	0.003712098
3 nM	0.048535	0.006189029
Control	0.05	0
