# Supplementary material for: The Organophosphate Paraoxon and Its Antidote Obidoxime Inhibit Thrombin Activity and Affect Coagulation In Vitro
Source: PLoS One. 2016 Sep 30;11(9):e0163787. doi: 10.1371/journal.pone.0163787 (PMC5045196; doi:10.1371/journal.pone.0163787)
Supplement: S3 Table — Average thrombin activity and standard deviation as calculated from thee different measurements of thrombin activity assay. (PDF) [file pone.0163787.s003.pdf]

S3 Table

Toxogonin	Average thrombin activity (U/ml)	Standard deviation
3 mM	0.000889679	4.30278E-05
1 mM	0.002025714	0.000110489
0.3 mM	0.007092	0.00326884
0.1 mM	0.008195429	0.000330065
30 nM	0.011049	0.000262821
3 nM	0.0106095	0.001765729
Control	0.05	0
