# Supplementary material for: The Organophosphate Paraoxon and Its Antidote Obidoxime Inhibit Thrombin Activity and Affect Coagulation In Vitro
Source: PLoS One. 2016 Sep 30;11(9):e0163787. doi: 10.1371/journal.pone.0163787 (PMC5045196; doi:10.1371/journal.pone.0163787)
Supplement: S4 Table — Average thrombin activity and standard deviation as calculated from six different measurements of thrombin activity assay. (PDF) [file pone.0163787.s004.pdf]

S4 Table

Atropine	Average thrombin activity (U/ml)	Standard deviation
0.9 mM	0.049378125	0.007271194
0.3 mM	0.044145833	0.003230137
90 uM	0.046875	0.004767894
30 uM	0.04574375	0.003381724
9 uM	0.04570625	0.001812252
3 uM	0.04475	0.003388895
Control	0.0500625	0.005264482
