# Supplementary material for: The Organophosphate Paraoxon and Its Antidote Obidoxime Inhibit Thrombin Activity and Affect Coagulation In Vitro
Source: PLoS One. 2016 Sep 30;11(9):e0163787. doi: 10.1371/journal.pone.0163787 (PMC5045196; doi:10.1371/journal.pone.0163787)
Supplement: S5 Table — Average times and standard deviation as calculated from three different measurements. (PDF) [file pone.0163787.s005.pdf]

S5 Table

	Control	Paraoxon 0.5 mM	Toxogonin 30 mM	Paraoxon 0.5 mM + Toxogonin 30 mM
Average TT (s)	16.9	16.975	67.325	82.925
Standard deviation	0.993310962	0.248746859	3.285098933	2.362599204
PT (s)	12.13333333	12.075	15.425	16.6
Standard deviation	0.169967317	0.147901995	0.286138079	0.3082207
APTT (s)	29.66666667	31.125	80.65	104.375
Standard deviation	1.837268504	0.227760839	3.228389691	5.508799779
